# Analysis of *TTG1* function in *Arabis alpina*

**DOI:** 10.1186/1471-2229-14-16

**Published:** 2014-01-10

**Authors:** Divykriti Chopra, Heike Wolff, Johannes Span, Swen Schellmann, George Coupland, Maria C Albani, Andrea Schrader, Martin Hülskamp

**Affiliations:** 1 Botanical Institute, Biocenter, Cologne University, Zülpicher Straße 47b, 50674 Cologne, Germany; 2 Max Planck Institute for Plant Breeding, Carl-von-Linne-Weg 10, 50829 Cologne, Germany

**Keywords:** Arabis alpina, TTG1, Trichomes, Root hairs, Pro-anthocyanidin, Anthocyanidin, Seed coat mucilage

## Abstract

**Background:**

In *Arabidopsis thaliana* (*A. thaliana*) the WD40 protein TRANSPARENT TESTA GLABRA1 (TTG1) controls five traits relevant for the adaptation of plants to environmental changes including the production of proanthocyanidin, anthocyanidin, seed coat mucilage, trichomes and root hairs. The analysis of different Brassicaceae species suggests that the function of TTG1 is conserved within the family.

**Results:**

In this work, we studied the function of *TTG1* in *Arabis alpina* (*A. alpina*). A comparison of wild type and two Aa*ttg1* alleles revealed that Aa*TTG1* is involved in the regulation of all five traits. A detailed analysis of the five traits showed striking phenotypic differences between *A. alpina* and *A. thaliana* such that trichome formation occurs also at later stages of leaf development and that root hairs form at non-root hair positions.

**Conclusions:**

The evolutionary conservation of the regulation of the five traits by TTG1 on the one hand and the striking phenotypic differences make *A. alpina* a very interesting genetic model system to study the evolution of TTG1-dependent gene regulatory networks at a functional level.

## Background

One approach towards a mechanistic understanding of phenotypic changes is evolutionary developmental biology (also called Evo-Devo) [1]. As most of our knowledge is based on a few well-characterized model systems that are separated by large evolutionary distances, evolutionary comparisons are often descriptive and have little functional depth. Typically Evo-Devo approaches aim to characterize the key players or pathways known to be relevant for a given process in one model organism in an evolutionarily distantly related species. As outlined by Sommer [2] this often leads to an almost descriptive list of the molecular inventories rather than a functional understanding. For a functional evolutionary comparison of developmental processes it is necessary to study clearly homologous processes in closely related species. This enables the understanding of changes in the regulatory network at a mechanistic level.

We focused on the *TTG1*-dependent gene regulatory network that is well-described in *A. thaliana*. Here it controls five traits that all have an adaptive value for the plant and are likely to be variable on the one hand, but also interdependent as they are controlled by the same regulatory genes [3]. TTG1 encodes a WD40 protein [4]. In *A. thaliana* TTG1 acts together with R2R3-MYB and bHLH proteins (called MBW complex) to regulate different aspects of epidermal cell differentiation including the production of proanthocyanidin, anthocyanidin, seed coat mucilage, trichomes and root hairs [5-12]. The bHLH factor is represented by three homologous, partially redundant acting genes. *TT8* regulates seed coat mucilage production, seed coat pigment production and anthocyanin biosynthesis. *EGL3* controls seed coat pigmentation, anthocyanin biosynthesis, trichome and root hair development and *GL3* is involved in anthocyanin biosynthesis, trichome and root hair development. High trait specificity is found for the R2R3-MYB factors such that one specific R2R3-MYB gene regulates each trait [3]. GL1 regulates trichome initiation [13], WER the non-root hair development [14], PAP1 and PAP2 anthocyanidin production [15,16], TT2 pro-anthocyanidin production and MYB61 regulates seed coat mucilage production [17]. During trichome and root hair development additional R3 single repeat MYBs are important as negative regulators mediating cellular interactions during pattern formation [18-24].

The function of the MBW complex in epidermal cell differentiation is evolutionary conserved in plants, though their regulation of anthocyanin and proanthocyanidin production seems to be the most ancient function. This is suggested by the finding that the MBW complex in maize is only involved in anthocyanin production [25,26], in petunia in anthocyanidin and proanthocyanidin production [27-29] and in *A. thaliana* in all five traits [6,7]. Based on the phylogenetic tree of the MYB proteins, Serna and Martin suggested that the additional role of the MBW complex in trichome formation has been adopted after the Asterid-Rosid division [5]. This view is supported by the findings that GL1 (*A. thaliana*) or C1 (*Zea mays*) overexpression in tobacco has no effect on trichome formation [30]. Conversely, overexpression of *TTG1* homologs from various species has been successfully used to complement the corresponding *A. thaliana* mutant phenotypes. These include *AN11* from *Petunia hybrida*[27], *PAC1* from maize [31], Gh*TTG1* and Gh*TTG3* from *Gossypium hirsutum* ([32]*,* In*WDR1/Ca* from *Ipomoea nil*[33], Mt*WD40-1* from *Medicago truncatula* ([34], Md*TTG1* from *Malus domestica*[35], Vv*TTG1* from *Vitis vinifera* L [36], and Pg*WD40* from *Punica granatum L*. [37]. This indicates that the biochemical function of TTG1 is functionally conserved over a large evolutionary distance.

Apart from *A. thaliana*, genetic data are available for two other species within the Brassicaceae family. In *Brassica rapa* it was shown that two traits, glabrous and yellow seeds, strictly co-segregated and that these two traits map to the Br*TTG1* locus [38]. In addition a yellow seed mutation was mapped to the Br*TT8* locus suggesting that also the function of the corresponding bHLH factor is conserved. In *Matthiola incana* it was shown that a line displaying white flowers, yellow seeds, seed mucilage defects and a glabrous phenotype exhibits a relevant point mutation in the Mi*TTG1* gene [39]. Together these data indicate that a function of TTG1 in the regulation of trichome, seed coat differentiation, anthocyanin and proanthocyanin pathways is conserved within the Brassicaceae.

As a complex gene regulatory network governs the regulation of the TTG1-dependent five traits it seems very attractive to study network evolution in this family. Towards this end it is desirable to systematically establish a second genetic model system enabling the functional characterisation by mutant analysis. We chose *A. alpina* for several reasons: On the one hand, *A. alpina* is sufficiently closely related to enable the identification of clear ortholog genes by sequence similarity and synteny in the fully sequenced genome. On the other hand the evolutionary distance of about 26 million years [40] to 40 million [41] between *A. thaliana* and *A. alpina* promised phenotypic variations for these traits and variations in the underlying gene regulatory networks. Finally, *A. alpina* can be transformed by Agrobacterium mediated gene transfer [42].

In this work we studied the function of *TTG1* in *Arabis alpina* (*A. alpina*). We demonstrate that all five traits are affected in two Aa*ttg1* alleles in *A. alpina*. As considerable phenotypic variation was already observed for some of these traits between different members of the Brassicaceae family [43-45] we did a detailed phenotypic description of the five traits to provide a reference for future studies. Our analysis revealed striking differences in the case of trichome and root hair patterning in *A. alpina* as compared to *A. thaliana*.

## Results

### Identification of Aa*ttg1* mutants in *A. alpina*

**Figure 1:**
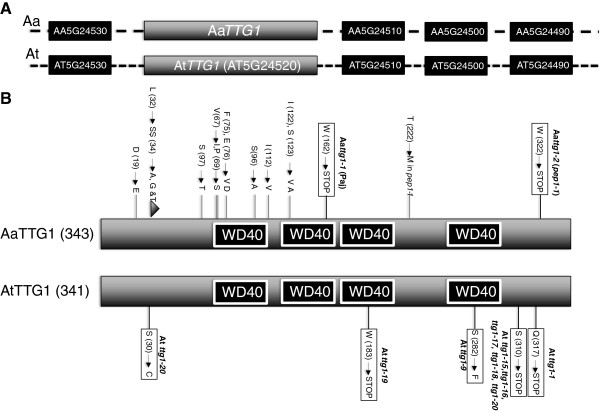
**Schematic presentation of Aa*TTG1 *and At*TTG1*. A)** Genomic regions of the *TTG1* loci including the neighboring genes in *A. alpina* and *A. thaliana*. The black boxes represent the homologous flanking genes. The gene names used for *A. alpina* are derived from the corresponding *A. thaliana* genes by exchanging the “At” for A. thaliana to “Aa” for *Arabis alpina*. As the distance between genes differs in the two species they are shown with different types of dashed lines. **B)** Schematic presentation of the protein structures. The position of the WD40 domains was determined using the ELM [46]. Differences of amino acids between *A. alpina* and *A. thaliana* are indicated. The amino acid position changed in mutant alleles in *A. alpina* (this work) and *A. thaliana*[4] have been marked by boxes.

To initiate a genetic dissection of TTG1-dependent regulation cascades in *A. alpina*, we identified the putative *A. alpina TTG1* gene by homology and synteny comparisons. The *A. alpina TTG1* gene (Aa*TTG1*) has a putative coding sequence of 1032 base pairs representing a putative protein of 343 amino acids. The Aa*TTG1* gene shows 90% sequence identity when compared to the *A. thaliana* At*TTG1* gene. The two flanking genes of Aa*TTG1* are homologs of those two flanking At*TTG1*: AT5G24530 and AT5G24510 (Figure 1A). This indicates that Aa*TTG1* and At*TTG1* are orthologs. A comparison of the amino acid sequence revealed differences in 12 amino acids and two additional amino acids (Figure 1B, Additional file 1: Table S1).

We identified two mutants in EMS mutagenized M2 populations of *A. alpina* showing a phenotype similar to *ttg1*: glabrous trichomes and yellow seeds. One mutant was recovered from the EMS treated wild-type background *A. alpina* Pajares (Paj) [42]. The other mutant was found in a mutagenized *A. alpina (*Paj) background carrying the *pep1-1* mutation [42,47,48]. In the *A. alpina (pep1-1*) background the *TTG1* gene contained a mutation that would lead to a T to M exchange at position 222 of the TTG1 protein, which, however, does not affect the function of TTG1 as this background shows no effect on any assumed TTG1-dependent phenotype. When sequencing the Aa*TTG1* gene in the *ttg1* mutant isolated from the Paj wild-type background we found a mutation leading to a stop codon after 161 amino acids in the second WD40 domain and called this allele Aa*ttg1-1*. The putative *ttg1* allele induced in the *pep1-1* background carried a mutation leading to a stop codon after 321 amino acids (called Aa*ttg1-2*). As a premature STOP codon two amino acids C-terminal to this position leads to strong *ttg1* phenotypes in *A. thaliana*[4], it is conceivable that also the *A. alpina* Aa*ttg1-2* allele shows the observed strong phenotypes (Figure 1B)*.* Together these data indicate that the two mutants identified by the trichome and seed color phenotypes are two *A. alpina* Aa*ttg1* alleles. We tested this by crossing A*attg1-1* and A*attg1-2* plants. F1 plants were glabrous confirming the allelism (Additional file 2: Figure S1A-E). To test whether AaTTG1 protein can rescue the Arabidopsis *ttg1* mutant phenotype we expressed the *Arabis alpina* coding sequence under the promoter of the Arabidopsis *TTG1* gene [49]. Towards this end we used the wild type coding sequence from *A. alpina* Pajares and *pep1-1*. We recovered 7 and four lines, respectively, displaying partial rescue of the trichome phenotype (Additional file 2: Figure S1F-H).

### (Pro-) anthocyanidin production in *A. alpina* wild type and Aa*ttg1* mutants

Brown colour of Arabidopsis seeds is caused by oxidized proanthocyanidins [50]. Screening for differences in seed pigmentation revealed a group of mutants with transparent testa - including *ttg1* - that was impaired in flavonoid accumulation [51,52]. Most of these genes were subsequently identified either as structural enzymes or regulators (e.g. TTG1) of the proanthocyanidin pathway [50].

**Figure 2:**
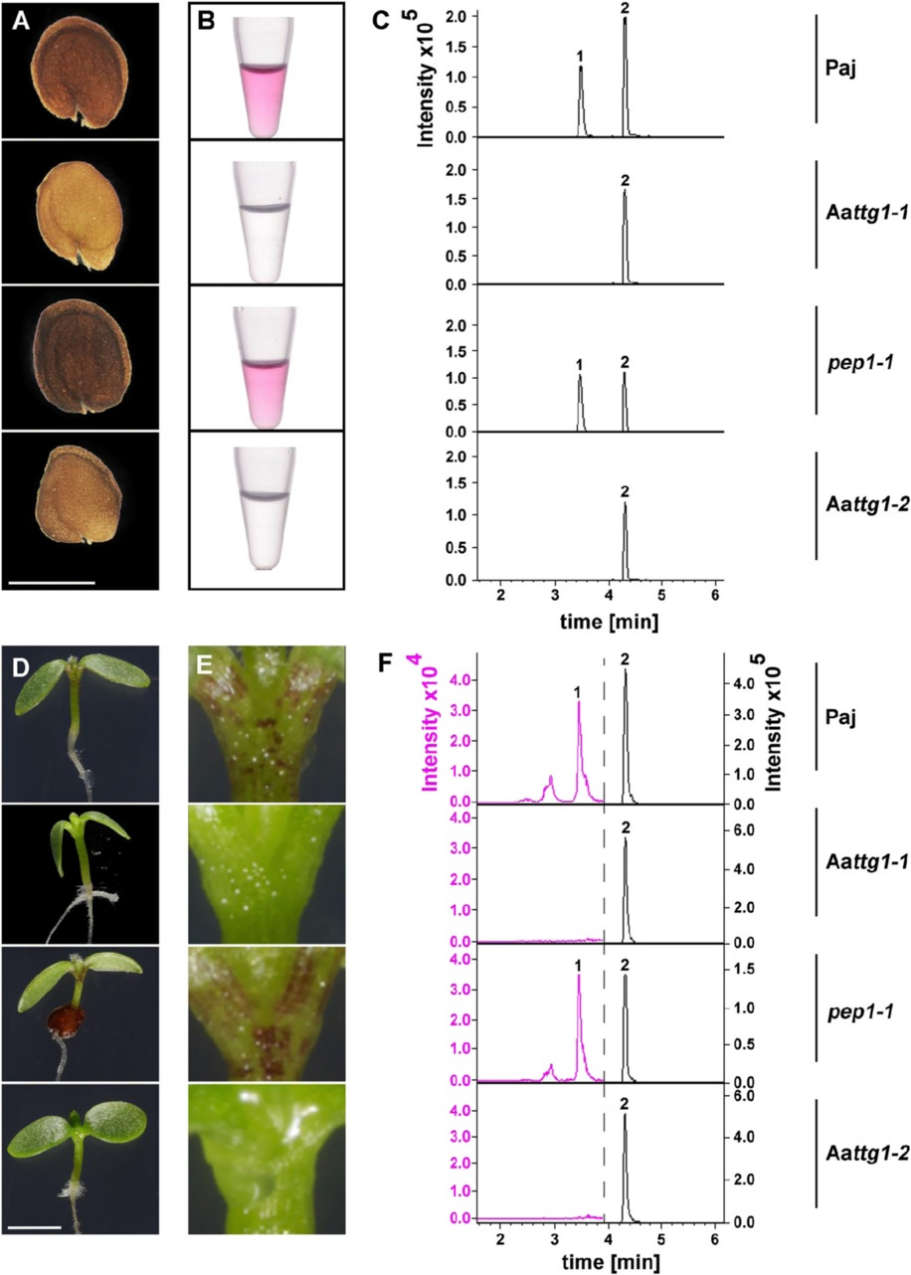
**TTG1 is needed for the accumulation of anthocyanidins in *A. alpina*.** Seeds **(A-C)** and seedlings **(D-F)** of Aa*ttg1-1* and Aa*ttg1-2* are devoid of cyanidin - a late component of the (pro-) anthocyanidin biosynthesis pathway - but contain kaempferol – an early component of this pathway. The genotype for each row in **A-C** and **D-F** is given on the right. **A)** stereo-microscopy of seeds. Aa*ttg1* mutants have a yellowish seed colour as compared to the respective backgrounds indicating a lack of proanthocyanidin; **B)** extracted and hydrolyzed insoluble components of the seeds‘ (pro-) anthocyanidin pathway, presence (backgrounds) or absence (Aa*ttg1* mutants) of pink colour relates to the presence or absence of (pro-) anthocyanidin in seeds; **C), F)** HPLC-MS analysis of extracted soluble, hydrolyzed components of the anthocyanidin biosynthesis pathway. Shown are extracted ion chromatograms for m/z = 287.055 +/- 0.005. Note that cyanidin and kaempferol have the same m/z value. Different scales were chosen to highlight the absence of cyanidin in **F)**. Full chromatograms are provided in Additional file 3: Figure S2. **D)** photography of 5 day-old seedlings grown on MS medium with 1% sucrose at constant light. **E)** zoom in on the petiole- and SAM-region of the seedlings shown on the left in **D)**. Aa*ttg1* mutants do not accumulate anthocyanidins in the seedling‘s hypocotyl. All pictures within one subfigure were taken at the same light settings. Bar in A: 1 mm; bar in C: 2 mm; 1: cyanidin (late biosynthesis compound); 2: kaempferol (early biosynthesis compound).

Compared to the corresponding backgrounds, the seed colour of both Aa*ttg1* mutants is yellowish, sharing the transparent testa phenotype with the mutants in *A. thaliana* (Figure 2A). This finding points to a lack of proanthocyanidins. The majority of procyanidin is found in the insoluble fraction of flavonoid extracts and can be analyzed by acidic hydrolysis to cyanidin [53,54]. We hydrolyzed the insoluble substances upon extraction for visual inspection (Figure 2B). A pink staining that is characteristic for cyanidin was obtained for the backgrounds but not for the Aa*ttg1* mutants. In subsequent HPLC-MS analysis of extracted soluble, hydrolyzed flavonoids no cyanidin was detectable, proofing its absence in Aa*ttg1* mutants (Figure 2C, Additional file 3: Figure S2). In contrast, kaempferol, was detected in the mutants and backgrounds, serving as a control for the successful extraction of flavonoids. This indicates that Aa*TTG1* either regulates the activity of the AaLDOX (leucoanthocyanidin dioxygenase) enzyme that catalyses the last step of cyanidin biosynthesis and/or earlier enzymes of the pathway.

The so called late genes of the anthocyanidin biosynthesis have been classified by their regulation through the TTG1 containing MBW complexes [3,55-59]. Therefore, our result identifies kaempferol as an early and cyanidin as a late component of the proanthocyanidin biosynthesis pathway in *A. alpina*.

The anthocyanidin biosynthesis pathway is part of the proanthocyanidin biosynthesis pathway. Red colour of *A. thaliana* hypocotyls and young leaves is the result of accumulating UV-protective anthocyanidins and their derivatives upon exposure to light or other stresses [60-62].

In *A. thaliana*, TTG1 as a general regulator also promotes visible accumulation of purple anthocyanins in the seedlings’ hypocotyls [3]. Our finding that Aa*ttg1* confers a transparent testa phenotype to *A. alpina* seeds suggests an absence of anthocyanins also in the hypocotyl. It is, however, possible that a redundant regulator exists for anthocyanin accumulation.

To address this, we grew seedlings of the Aa*ttg1* alleles and their respective backgrounds under constant light on plates supplemented with sucrose. We found that in the hypocotyls of Aa*ttg1* mutant seedlings no visible pink anthocyanin was accumulated in contrast to Paj and *pep1-1* (Figure 2D-E). Moreover, HPLC-MS analysis for both Aa*ttg1* mutants showed that Aa*TTG1* is also essential for the accumulation of cyanidin in seedlings and its function is not taken over by any other gene (Figure 2F, Additional file 3: Figure S2).

### Seed coat differentiation in *A. alpina* wild type and Aa*ttg1* mutants

**Figure 3:**
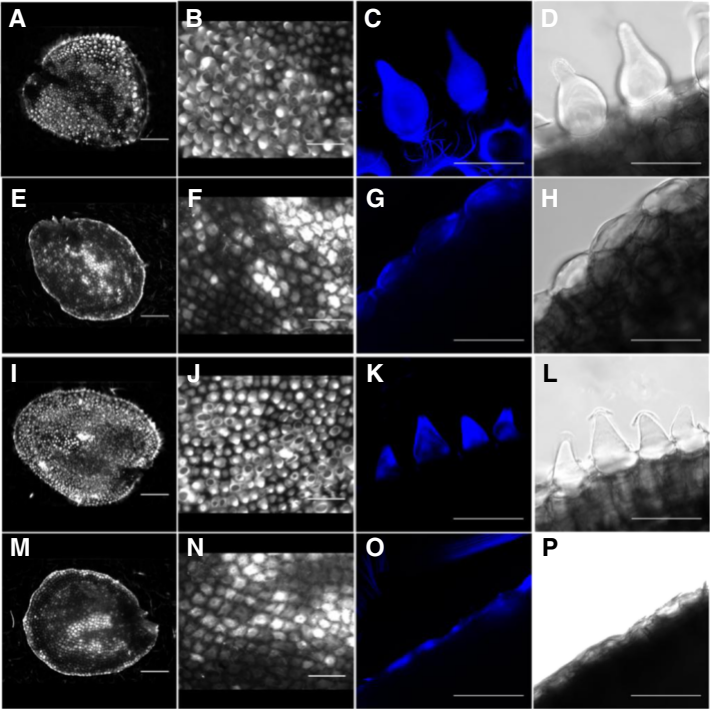
**Calcofluor white stained wild type and Aa*ttg1 *mutant seeds.** Fluorescence microscope **(A,B,E,F,I,J,M,N)** and CLSM (fluorescence channel: **C,G,K,O,** transmission channel: **D,H,L,P**) pictures of the surface of *A. alpina* seeds stained with calcofluor white. **A** to **D**) wild type Paj. **I** to **L**) *pep1-1* mutant. Note, that in wild type and *pep1-1* mutants the columella is seen as large domes. **E** to **H**) Aa*ttg1-1* mutant induced in the wild type Paj background. **M** to **P**) The Aa*ttg1-2* mutant induced in the *pep1-1* background. In both mutants only the rim of the epidermal cells is left and columellas are absent. Scale bar: **A**,**E**,**I**,**M** = 300 μm, **B**,**F**,**G**,**N** = 100 μm, **C**,**D**,**G**,**H**,**K**,**L**,**O**,**P** = 50 μm.

In *A. thaliana*, seed epidermal cells of the outer integument differentiate into highly specialized seed coat cells. These are characterized by the formation of a central column surrounded by a secondary cell wall (the columella) and the accumulation of polysaccharide mucilage between the plasma membrane and the primary cell wall that is released in the presence of water [63,64]. In *A. thaliana*, *ttg1* mutants neither form a columella nor produce mucilage [51]. We compared columella formation in the two *ttg1* mutants with the respective genetic backgrounds Paj and *pep1-1* by staining with calcofluor white (Figure 3), Ruthenium Red (Additional file 4: Figure S3) and by Scanning Electron Microscopy analysis (Additional file 5: Figure S4). Wild type and *pep1-1* seeds have an irregular but smooth surface with domes representing the columella (Figure 3A-D, I-L). In the two corresponding Aa*ttg1* mutants, the columella is completely missing indicating that epidermal differentiation of the seed coat is affected similar as observed in At*ttg1* mutants [51,63,64] (Figure 3E-H, M-P).

### Trichome patterning in *A. alpina* wild type and Aa*ttg1* mutants

**Figure 4:**
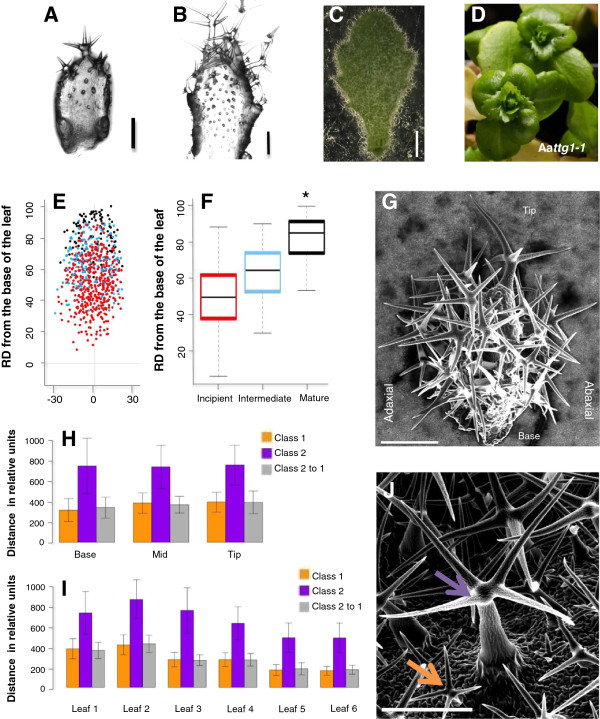
**Trichome patterning in *A. alpina*. A-C)** Three developmental stages of an *A. alpina* leaf. **D)** Aa*ttg1-1* leaves. **E)** Meta leaf generated using TrichEratops integrating the relative position of trichomes from 22 young leaves [66]. Red dots are incipient trichomes, blue dots intermediate developmental stages and black dots represent mature trichomes. Note that incipient trichomes are found in a region in which already mature trichomes have developed. **F)** Boxplot depicting the relative position (0 is the base of the leaf and 100 the tip of the leaf) of three developmental classes of trichome development. Note that the average position of the three developmental classes is clearly distinct. “*” indicate that all three classes are significantly different from each other according to Student’s t-test (*P* < 0.001). **G)** SEM picture of a leaf 3 from the side showing that trichomes are found on the abaxial and adaxial side. **H)** Average minimum distance to the nearest neighboring trichome on different regions of leaf 2. Distances between smaller trichomes (class 1) are shown in orange, distances between larger trichomes (class 2) in pink and distances between the two classes in grey. **I)** Average minimum distance to the nearest neighboring trichome on leaves 1 to 6. **J)** SEM of an adult *A. alpina* leaf showing the large (pink arrow) and the small classes of trichomes (orange arrow). Scale bar = 100 μm in **A,B**. Scale bar = 500 μm in **C**. Scale bar = 1 mm in **D,E**.

In *A. thaliana*, trichomes are initiated on young leaves and become separated by division and expansion of the epidermal cells lying in between [65]. At the first glance, trichome initiation on *A. alpina* leaves is very similar to the situation in *A. thaliana* (Figure 4A-C): At the base of young leaves, we found incipient trichomes. These comprise trichome stages preceding branch initiation (Figure 4A). Mature trichomes with several branches were found at the leaf tip and intermediate stages were observed in the mid region (Figure 4B). Adult leaves are covered densely with trichomes (Figure 4C). However, we noted one striking difference: on mature leaves we found two classes of mature trichomes that differed in size and height (Figure 4J). Our visual impression was that the larger trichomes (class 2) are arranged in a regular pattern with the smaller trichomes (class 1) being scattered between them (Figure 4J). To test this, we systematically measured the distances between the two classes of trichomes. We analyzed three regions of the third leaf: a region at the base, in the middle and the tip. In all three regions, on average the larger trichomes showed about twice the distance from each other as compared to the distances in between smaller trichomes and between smaller trichomes and the larger trichomes (Figure 4H). The same pattern was also found on the second to the sixths leaves indicating that it is a general feature (Figure 4I). These distance patterns suggested to us two superimposed trichome patterns. We therefore studied young leaf stages in more detail using the analysis tool TrichEratops [66]. A meta leaf was generated, in which the position of three developmental trichome stages are shown at their relative positions on the leaf with respect to the basal-distal axis (Figure 4E). As described in *A. thaliana*, we found a general gradient of trichome developmental stages with mature trichomes at the tip and young trichomes at the base of the leaf. In contrast to *A. thaliana*, we observed early developmental stages of trichome development between already mature trichomes (Figure 4B, F). These patterns may either be explained by the formation of new trichomes between more mature trichomes or by spatial differences in the growth characteristics such that the smaller trichomes are initiated normally, but grow slower. During the course of our experiments we noted that all *A. alpina* leaves are densely covered with trichomes on both the adaxial and the abaxial sides (Figure 4G). This is in contrast to *A. thaliana* where only late leaves produce abaxial trichomes as a consequence of the phase change from vegetative to generative growth [67]. To analyse the role of *A. alpina TTG1* in trichome formation we analysed the two *ttg1* mutants Aa*ttg1-1* and Aa*ttg1-2.* As known from strong *ttg1* mutants in *A. thaliana* both alleles lacked trichomes completely (Figure 4D).

### Root hair patterning in *A. alpina* wild type and Aa*ttg1* mutants

**Figure 5:**
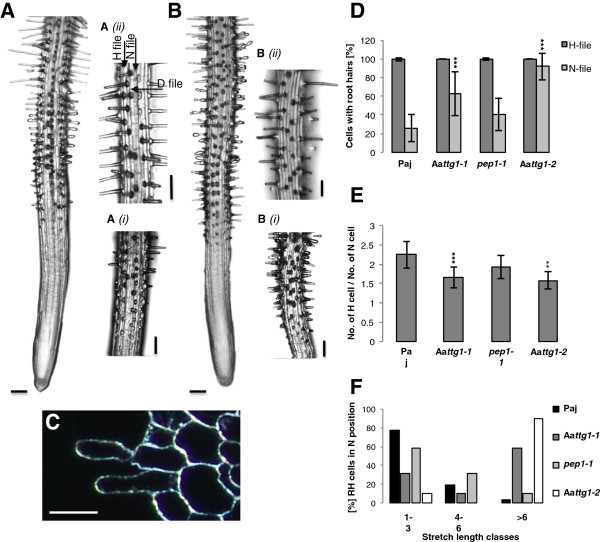
**Root hair patterning in *A. alpina*. A)** Wild type *A. alpina* root; **A(i)**, Higher magnification of the lower root hair differentiation zone; **A(ii)**, Higher magnification of upper root hair differentiation zone. Root hair file (H), Non-root hair file (N) and non-root hair file with root hair stretches (D-file) is indicated by arrows. **B)** Aa*ttg1-1* root; **B(i)**, Higher magnification of the lower root hair differentiation zone; **B(ii)**, Higher magnification of the upper root hair differentiation zone. **C)** Wild type *A. alpina* root cross section depicting one root hair at the H-position over the cleft of two underlying cortex cells and a neighboring root hair in an N-position. **D)** Percentage of root hairs in root hair files (H) and non-root hair files (N). **E)** Relative number of cells in H and N files. A ratio of about two reflects that the non-hair cells are twice as long as the H-cells. **F)** Number of cells in continuous stretches of root hair cells in the N files. Scale bar: 100 μm in **A,B**; Scale bar: 40 μm in **C**. ***: *P* < 0.001, **: *P* < 0.01.

In *A. thaliana*, epidermal root cells are arranged in files. Cells overlaying the cleft between two underlying cortex cells are short and differentiate into hair cells (H–file) [68]. Cells in all other files are long and do not develop root hairs (called N-files). To understand root hair patterning in *A. alpina*, we analysed file-specific root hair production in two regions of the differentiation zone to determine the temporal spatial development of the root hair pattern (Figure 5A). Both, *A. alpina* Paj and *pep1-1* showed a similar frequency of root hair cells in the lower (Figure 5Ai) and in the upper region (Figure 5Aii) indicating that the final root hair pattern is determined already in the lower region (data not shown). Similar to *A. thaliana* almost all cells in the H–position develop into root hairs. However, in *A. alpina* cells in N-file positions also frequently form root hairs (Figure 5A, C, D). We found between 30% (Paj) and 40% (*pep1-1*) of the N-position cells to develop into root hairs (Figure 5D). This finding raised the question, whether the cells in the H– and N-positions also differ in other characteristics. One difference reported in *A. thaliana* is the length of the individual cells such that cells in N-files are about twice as long as H–file cells [69]. As the cell length continuously changes along the root axis due to cell growth, we did not measure the actual cell lengths, but rather determined the H- to N-file cell number ratios. In *A. alpina* Paj we found a ratio of 2.3, in *pep1-1* the ratio was 2.0, indicating that the N-file cells are approximately twice as long as H-file cells (Figure 5E). During the course of experiments we noticed that the cells carrying a root hair in N position were not evenly distributed along the file, but were arranged in continuous stretches of N-file cells differentiating into root hairs. As depicted in Figure 5F, more than 50% of all stretches consisted of 1 to 3 cells, indicating a higher probability for short (1–3) than for long (>6) stretches. Typically, we found root hairs in N-position always on only one side of an H-file. Out of 542 Paj H-file cells, just one was flanked symmetrically by two N-file cells carrying a root hair (*pep1-1*: 20 out of 511). To study the function of *A. alpina TTG1* in root hair patterning we studied root hair patterning in Aa*ttg1-1* and Aa*ttg1-2.* We found ectopic root hairs at N-positions similar as described in *A. thaliana* (Figure 5B, D).

## Discussion

TTG1 containing MBW complexes regulate five different traits in *A. thaliana* including proanthocyanidin, anthocyanidin and mucilage production as well as trichome and root hair patterning. Taking together the data on single or multiple TTG1-dependent traits studied in different Brassicaceae species indicates that *TTG1* has the same range of functions as described in *A. thaliana* throughout this family. For *Brassica rapa* a function in trichome and seed color was reported [38]. In *Matthiola incana* a role in (pro)-anthocyanin, mucilage and trichome formation was shown [39]. In support of these findings, we show that these four traits and in addition root hair patterning are affected in two independent Aa*ttg1* alleles in *A. alpina*.

These five traits have an adaptive value for the plant. Therefore, on the one hand, variability between species might be expected. On the other hand, these traits are regulated by differential TTG1 complexes encoded by members of the TTG1 gene regulatory network. Due to this genetic interdependence variability might be limited. In support of these considerations we found no apparent differences between *A. alpina* and *A. thaliana* for some but not all traits.

According to Serna and Martin, the TTG1-dependent regulation of (pro-) anthocyanidin production is a more ancient trait than trichome formation [5]. In agreement with this, the TTG1-dependent definition of early and late substances between kaempferol and cyanidin is maintained in *A. alpina* as compared to *A. thaliana*[3,55-59]. Similarly, AaTTG1 is needed for columella formation as described for *A. thaliana*[51].

While the phenotypes of these traits are generally very similar to those in *A. thaliana*, we noted some striking deviations for trichome and root hair patterning that are not trivial to explain in the light of the known regulation schemes. The observed two superimposed trichome patterns can be explained in two ways. One possibility is that small and large trichomes are initiated around the same time and that the subsequent cell differentiation differs. In this scenario, genes regulating cell differentiation and/or morphogenesis would be differentially expressed in the two types of trichomes. This could occur at different levels including genes regulating differentiation such as MYB5 and MYB23 [70], GL2 [71,72], TTG2 [73] or genes controlling endoreduplication such as for example the group of KAK genes [74]. The second possibility is that trichome initiation occurs in developmentally advanced stages of leaf development*.* In *A. thaliana* this phenotype was found in lines overexpressing the GL3/EGL3 homolog from maize, the R-gene [75]. By analogy, the intercalation phenotype in *A. alpina* could be explained by changes in the spatial/temporal expression of *A. alpina GL3*. The formation of root hairs in N-files is also not found in wild type *A. thaliana* under normal conditions but is reminiscent of At*ttg1* and At*wer* mutants [14,76]. Thus, one possibility to derive the wild-type pattern in *A. alpina* roots is a reduction of the Aa*TTG1* or Aa*WER* activity. Given that mutations reducing trichome number lead to the production of additional root hairs because the MBW complex serves to activate trichomes and non-root hair fates the production of additional root hairs and extra trichome formation between mature trichomes cannot easily be explained by the *A. thaliana* network.

## Conclusions

Taken together, our results demonstrate that the phenotypes of the five *TTG1*-dependent traits studied here are in general very similar in *A. thaliana* and *A. alpina.* The various phenotypic differences make *A. alpina* a very interesting genetic model system to study the evolution of gene regulatory networks at a functional level.

## Methods

### Plant Material and growth conditions

The Aa*ttg1* alleles were isolated from EMS mutagenized *A. alpina* Pajares [42] and *pep1-1* populations. For root hair file analysis, seeds were surface sterilized with 70% (v/v) ethanol (5 min) and 2% sodium hypochlorite (w/v, 8 min). Sterilized seeds were sown on 1× Murashige-Skoog plates lacking sucrose and stratified at 4°C for 5 days. Plants were grown on vertically positioned plates for 7 days under long day (LD) conditions (16 h light, 8 h darkness) at 21°C. For inter-species rescue experiments we used the Arabidopsis *ttg1-1* mutant (L*er* background, [77]).

For soil-grown *A. alpina*, seeds were stratified in darkness at 4°C for four days and then placed in growth chambers under LD conditions at 21°C.

### Sequence and synteny analysis

Extracted Aa*TTG1* (Paj and *pep1-1*) sequences were analysed on CLC DNA Workbench 5.6.1 (CLC bio, Aarhus, Denmark) by comparison with CDS of AT5G24520 downloaded from TAIR (http://www.arabidopsis.org). NCBI Blastn 2.2.28 was used to confirm the synteny of the neighbouring genes [78]. Primers rev_ttg1_arabis_out (5‘-GCAATCAAGAATCTCTAGAACCAAG-3‘) and fwd_ttg1_arabis_out (5’-CAAATGTATGGACCGAATTATCAAG-3‘) were designed outside the CDS of Aa*TTG1* to sequence it from the wild types and the mutants.

### Trichome analysis

The first true leaf of soil-grown *A. alpina* was labeled as leaf 1 and the following ones accordingly. When leaf 6 had reached a size of approximately 2 cm in length, leaves 1–6 were used for trichome analysis. For this, all leaves of one developmental stage were photographed at a magnification that enabled the distinction of different trichome classes. The pictures where analyzed using the TrichEratops software by marking the two different classes of trichomes [66]. The distance between trichomes of one class and trichomes of different classes were calculated with R (http://www.r-project.org/). Young leaves were essentially analyzed as described previously [66].

### Root hair file analysis

H-files of 7-day-old plate-grown seedlings were microscopically identified by the position over cortical cell boundaries. Following 10 to 15 H-file cells per root and zone, the number of cells and root hairs in the flanking N-files was determined. Further, the length of continuous stretches of N-file cells carrying a root hair was determined. For cross-sections, all root tissues were fixed and embedded as previously described [79]. Agarose was used for mechanical fixation. 100 μm sections were made using a Leica EM UC7 ultra microtome (Leica Microsystems, Wetzlar, Germany) with glass knives.

### Flavonoid analysis

For extraction of flavonoids, seeds were homogenized in 100 μl MeOH/water/formic acid (50:49:1, v/v) with glass beads in a tissue lyser (Qiagen, Hilden, Germany) at 30 Hz for 180 sec. Following centrifugation, the pellet was extracted with 200 μl MeOH/water/formic acid (50:49:1, v/v) over night at 4°C. 60 μl of centrifuged pooled supernatants were mixed with 440 μl MeOH:HClconc. (95:5, v/v), hydrolyzed for 90 min and diluted 1:1 with MeOH prior to LC-MS analysis. For photography, pellets were treated in the same way with 200 μl MeOH:HClconc. (95:5, v/v). For seedlings, 200 μl of MeOH:HClconc. (95:5, v/v) were used for homogenization and over night extraction followed by direct hydrolysis.

Mass analysis was done with a Dionex 3000 UPLC (Thermo Scientific, Dreiech, Germany) - maXis 4G (Bruker Daltonics, Bremen, Germany) LC-MS system equipped with an Apollo II ESI source (Bruker Daltonics, Bremen, Germany). 5 μl of samples were separated with a Poroshell 120, EC-C18, (3×50 mm, 2.7 μm) C18 column (Agilent, Waldbronn, Germany) and mix of solvent A water (0.1% formic acid) and solvent B MeOH (0.1% formic acid) with gradient profile (starting with 95:5, v/v, for 0.5 min; linear gradient up to 0:100, A/B, over 3.9 min and maintained for 2 min and re-equalibration to 95:5, A/B with a total runtime of 8.6 min) at a flow rate of 0.3 ml/min. LC-MS analysis, data processing and annotation of kaempferol and cyanidin were carried out with Compass DataAnalysis Version 4.0 SP5 (Bruker Daltonics, Bremen, Germany). Metabolites were identified by comparison to kaempferol and cyanidin (Sigma, Germany).

### Constructs and transformation

The binary vector _pro_At*TTG1*pAMPAT-GW [49] was used to create _pro_At*TTG1:*Aa*TTG1*(Paj) and _pro_At*TTG1:*Aa*TTG1*(*pep1-1*) using the Gateway^R^ system (Invitrogen) using the following primers for the cloning of Aa*TTG1*: Fwd: GGGGACAAGTTTGTACAAAAAAGCAGGCTTAATGGATAACTCAGCTCCAGA.

Rev: GGGGACCACTTTGTACAAGAAAGCTGGGTTTCAAACTCTAAGGAGCTGCA. The constructs were introduced in the *A. thaliana ttg1-1* mutant (L*er* background, [77]) by Agrobacterium-mediated (strain GV3101-pMP90RK) transformation using the floral dip method described previously [80]. Transformants were selected in the T1 generation on soil by screening for trichomes on leaf number 3 or 4.

### Photography and microscopy

Whole leaves were captured using a Canon EOS 5D Mark (Canon, Krefeld, Germany). Dry *A. alpina* seeds were mounted on a conductive carbon tab covered SEM stub and analyzed using a FEI Quanta FEG 250 Scanning Electron Microscope (SEM; FEI, Eindhoven, The Netherlands) at an accelerating voltage of 15 kV. *A. alpina* seeds were stained with 0.01% (w/v) aqueous solution of calcofluor white (Fluroscent Brightner, Sigma-Aldrich, Germany) overnight and analysed by creating manual z-stacks with Leica DM5000B microscope fitted with a LEICA DFC 360 FX camera and a Leica LAS AF software (Leica Microsystems, Wetzlar). Optical sections of the calcofluor white stained seeds were obtained by confocal laser scanning (CLSM) microscopy using the Leica TCS SPE CLSM, Leica LCS software (Leica Microsystems, Wetzlar). 0.05%(w/v). Aqueous ruthenium red (Sigma-Aldrich, Germany) stained seeds (overnight) were analysed with a conventional light microscope, Leica DMRE microscope, Leica LCS software (Leica Microsystems, Wetzlar). Root hair files were analyzed by creating z-stacks by conventional light microscopy using a Leica DM5000B fluorescence microscope (Leica Microsystems, Wetzlar). Images were processed using ImageJ (Rasband, W.S., ImageJ, U.S. National Institutes of Health, Bethesda, Maryland, USA, http://imagej.nih.gov/ij/, 1997–2012) and Photoshop 7.0.1, Adobe. Pictures of seeds (Figure 2) and of the adult leaf (Figure 4C) were acquired using a Leica stereomicroscope (MZ FLIII) with the MultiFocus and Montage option of the Leica Application Suite V3 (Leica Microsystems, Wetzlar, Germany).

### Statistical analysis and software

Statistical analysis was done as described before [81]. We used the Tricharatops [66] and R software (http://www.r-project.org/) to create the meta leaf, box plots and graphs for the analysis of the minimum distance to the nearest neighbouring trichome. Microsoft Excel (Microsoft, Redmond, USA) was used for the diagrams analyzing the root hair pattern.

## Authors’ contributions

DC, HW, JS, SS, MCA AS performed the experiments, GC, AS and MH supervised the project, AS and MH wrote the manuscript. All authors read and approved the final manuscript.

## Supplementary Material

Additional file 1: Table S1 Comparison of amino acid (aa) sequences between AtTTG1 and AaTTG1. Click here for file

Additional file 2: Figure S1 Complementation test between the Aa*ttg1-1* and Aa*ttg1-2* mutant alleles and rescue experiments in *A. thaliana.* First true leaves of A) wild type Paj, B) Aa*ttg1-1*, C) *pep1-1*, D) Aa*ttg1-2*, E) First true leaf of a F1 plant from the cross between Aa*ttg1-1* and Aa*ttg1-2*. Leaves are glabrous indicating allelism. Scale bar = 1 mm. F) *A. thaliana ttg1-1* rosette leaves. Plants are completely devoid of trichomes in this allele. G) *A. thaliana* At*ttg1-1*_Pro_At*TTG1*:Aa*TTG1*^Paj^ plant showing partial rescue of the trichome phenotype. H) At*ttg1-1*_Pro_At*TTG1*:Aa*TTG1*^*pep1-1*^ plant showing partial trichome rescue. Scale bar = 1 mm. Click here for file

Additional file 3: Figure S2 HPLC-MS analysis of cyanidin and kaempferol in seeds and seedlings of *A. alpina* – full chromatograms. Shown are total ion chromatogram (TIC, left) and extracted ion chromatogram (EIC, right) for 750 nM cyanidin (MeOH), 750 nM kaempferol (MeOH) and for all samples shown in Figure 2C and Figure 2F. The m/z value for cyanidin ([M]+) and kaempferol ([M + H]+) is 287.055. EICs for m/z = 287.055 +/− 0.005 were generated based on the corresponding TICs using the Compass DataAnalysis software Version 4.0 SP5 (Bruker Daltonics, Bremen, Germany). In the TIC the retention time and in the EIC the peak of cyanidin and kaempferol is marked. 1: cyanidin; 2: kaempferol. Dashed lines mark the begin and end of each sample. Before the first dashed line the mass calibration for each run can be seen in the TIC. Click here for file

Additional file 4: Figure S3 Ruthenium red stained seeds of wild type and Aa*ttg1* mutants. Light microscopy image of the surface of *A. alpina* seeds. The dome shaped columella is stained with ruthenium red labeling the seed coat mucilage. A) Wild type Paj. B) *pep1-1* mutant. C) Aa*ttg1-1* mutant induced in the wild type Paj background. D) Aa*ttg1-2* mutant induced in the *pep1-1* background. Note, the absence of ruthenium red stained columellas in both mutants. Scale bar = 500 μm, inset = 50 μm. Click here for file

Additional file 5: Figure S4 SEM pictures of wild type and Aa*ttg1* mutant seeds. Scanning Electron Micrographs of the surface of *A. alpina* seeds. A, B) wild type Paj and *pep1-1* mutant, respectively. Note, that the surface is irregularly but smooth and that the columella is seen as small domes. C, D) Aa*ttg1-1* mutant induced in the wild type Paj background and the Aa*ttg1-2* mutant induced in the *pep1-1* background. Only the rim of the epidermal cells is left. Columellas are absent. Scale bar: 100 μm. Click here for file
