# Supplementary material for: Analysis of TTG1 function in Arabis alpina
Source: BMC Plant Biol. 2014 Jan 10;14:16. doi: 10.1186/1471-2229-14-16 (PMC3904473; doi:10.1186/1471-2229-14-16)
Supplement: Additional file 1: Table S1 — Comparison of amino acid (aa) sequences between AtTTG1 and AaTTG1. [file 1471-2229-14-16-S1.pdf]

Table S1: Comparison of amino acid (aa) sequences between AtTTG1 and AaTTG1

**aa exchange**	**aa position in *A. alpina***	**aa position in *A. thaliana***
D to E	19	19
L to S, S to AGT	32, 34, 35, 36	32, 34
S to T	59	57
V to I	67	65
P to S	69	67
F to V	75	73
E to D	76	74
S to A	96	94
I to V	112	110
I to V	122	120
S to A	123	121
