# Supplementary figures and images for: Analysis of TTG1 function in Arabis alpina
Source: BMC Plant Biol. 2014 Jan 10;14:16. doi: 10.1186/1471-2229-14-16 (PMC3904473; doi:10.1186/1471-2229-14-16)

## Slide 1
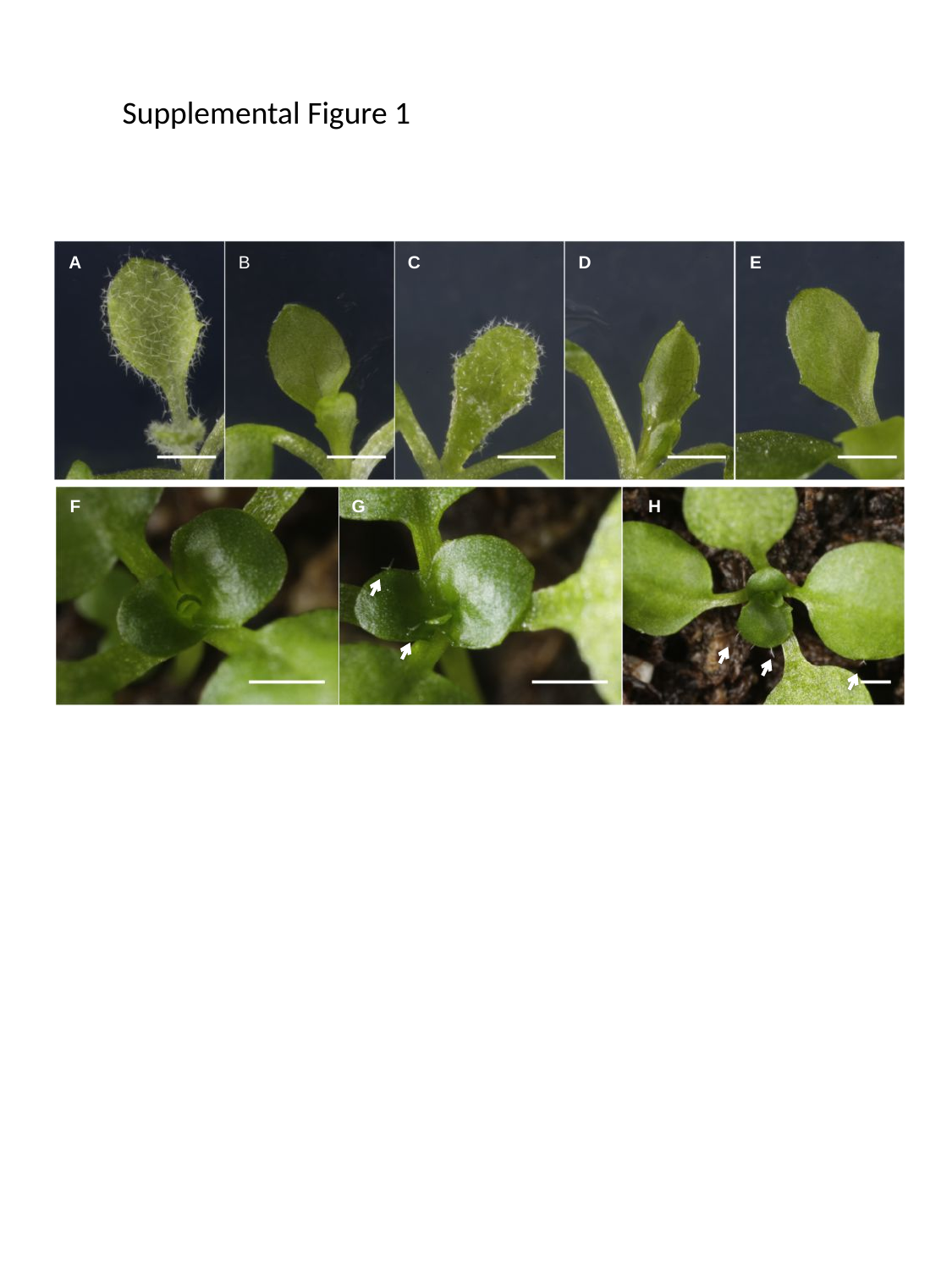

Supplemental Figure 1
A
B
C
D
E
F
G
H

Supplement: Additional file 2: Figure S1 — Complementation test between the Aattg1-1 and Aattg1-2 mutant alleles and rescue experiments in A. thaliana. First true leaves of A) wild type Paj, B) Aattg1-1, C) pep1-1, D) Aattg1-2, E) First true leaf of a F1 plant from the cross between Aattg1-1 and Aattg1-2. Leaves are glabrous indicating allelism. Scale bar = 1 mm. F) A. thaliana ttg1-1 rosette leaves. Plants are completely devoid of trichomes in this allele. G) A. thaliana Atttg1-1ProAtTTG1:AaTTG1Paj plant showing partial rescue of the trichome phenotype. H) Atttg1-1ProAtTTG1:AaTTG1pep1-1 plant showing partial trichome rescue. Scale bar = 1 mm. [file 1471-2229-14-16-S2.pptx]

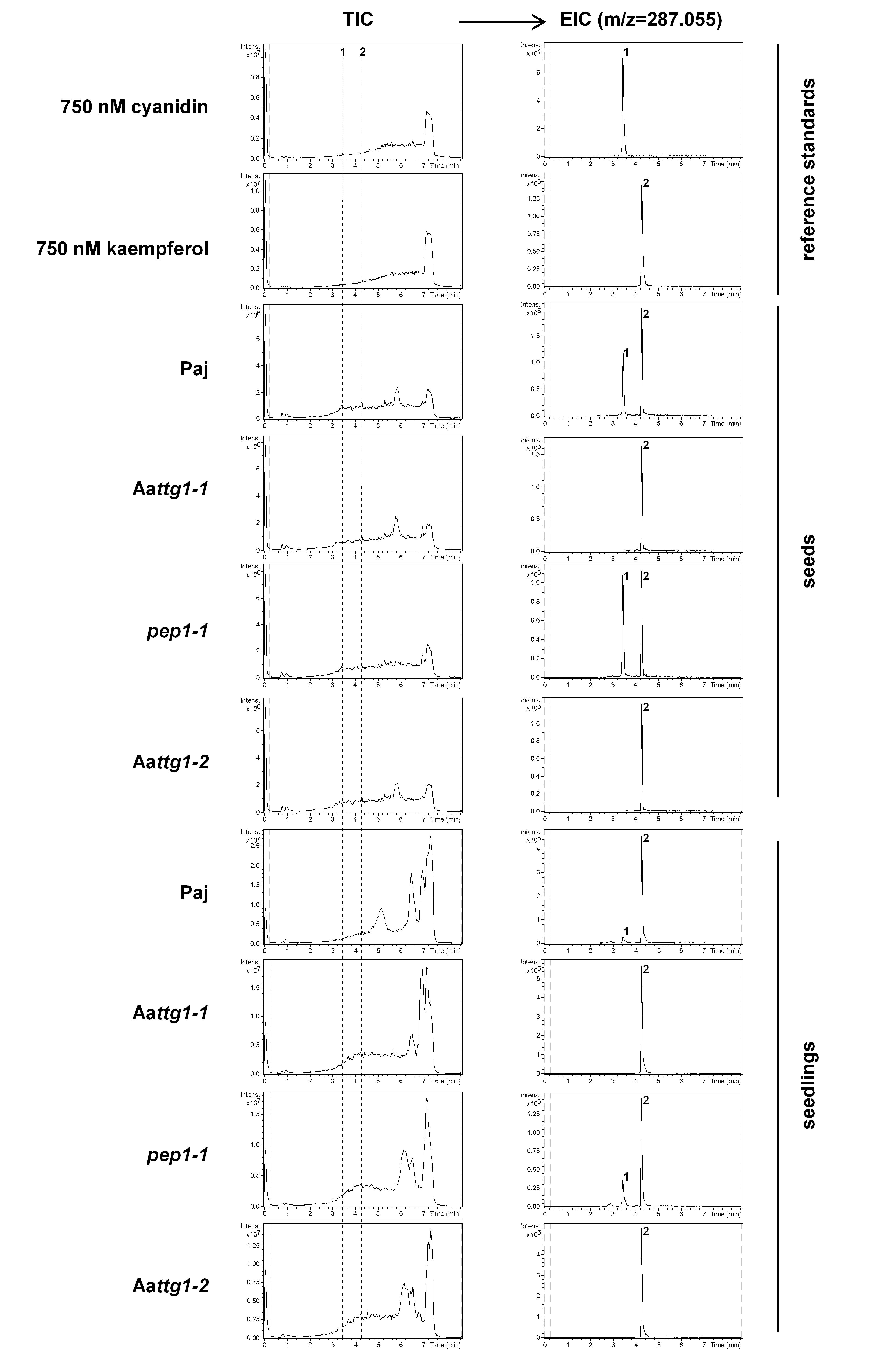

Supplement: Additional file 3: Figure S2 — HPLC-MS analysis of cyanidin and kaempferol in seeds and seedlings of A. alpina – full chromatograms. Shown are total ion chromatogram (TIC, left) and extracted ion chromatogram (EIC, right) for 750 nM cyanidin (MeOH), 750 nM kaempferol (MeOH) and for all samples shown in Figure 2C and Figure 2F. The m/z value for cyanidin ([M]+) and kaempferol ([M + H]+) is 287.055. EICs for m/z = 287.055 +/− 0.005 were generated based on the corresponding TICs using the Compass DataAnalysis software Version 4.0 SP5 (Bruker Daltonics, Bremen, Germany). In the TIC the retention time and in the EIC the peak of cyanidin and kaempferol is marked. 1: cyanidin; 2: kaempferol. Dashed lines mark the begin and end of each sample. Before the first dashed line the mass calibration for each run can be seen in the TIC. [file 1471-2229-14-16-S3.jpeg]

## Slide 1
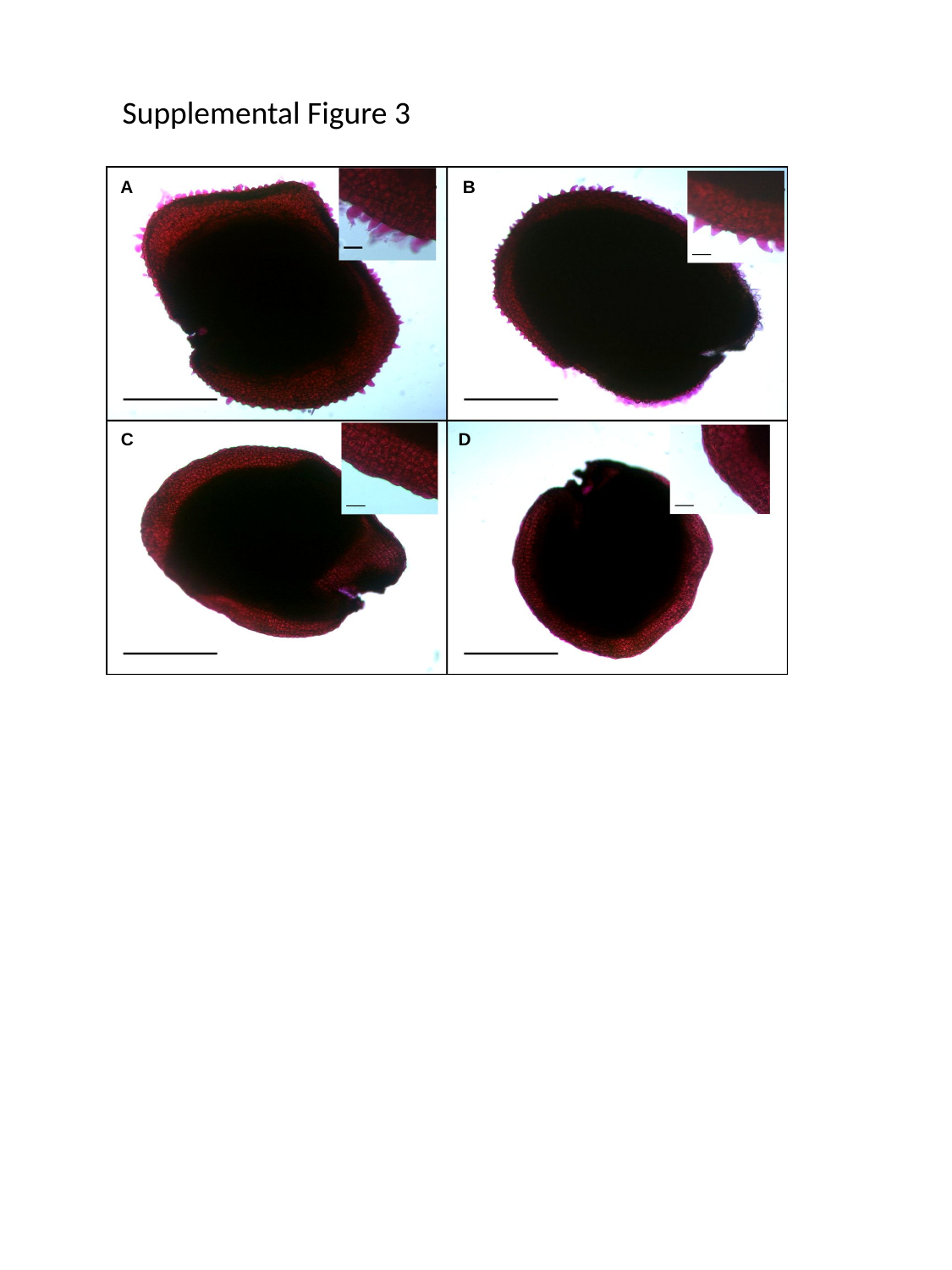

Supplemental Figure 3
A
B
D
C

Supplement: Additional file 4: Figure S3 — Ruthenium red stained seeds of wild type and Aattg1 mutants. Light microscopy image of the surface of A. alpina seeds. The dome shaped columella is stained with ruthenium red labeling the seed coat mucilage. A) Wild type Paj. B) pep1-1 mutant. C) Aattg1-1 mutant induced in the wild type Paj background. D) Aattg1-2 mutant induced in the pep1-1 background. Note, the absence of ruthenium red stained columellas in both mutants. Scale bar = 500 μm, inset = 50 μm. [file 1471-2229-14-16-S4.pptx]

## Slide 1
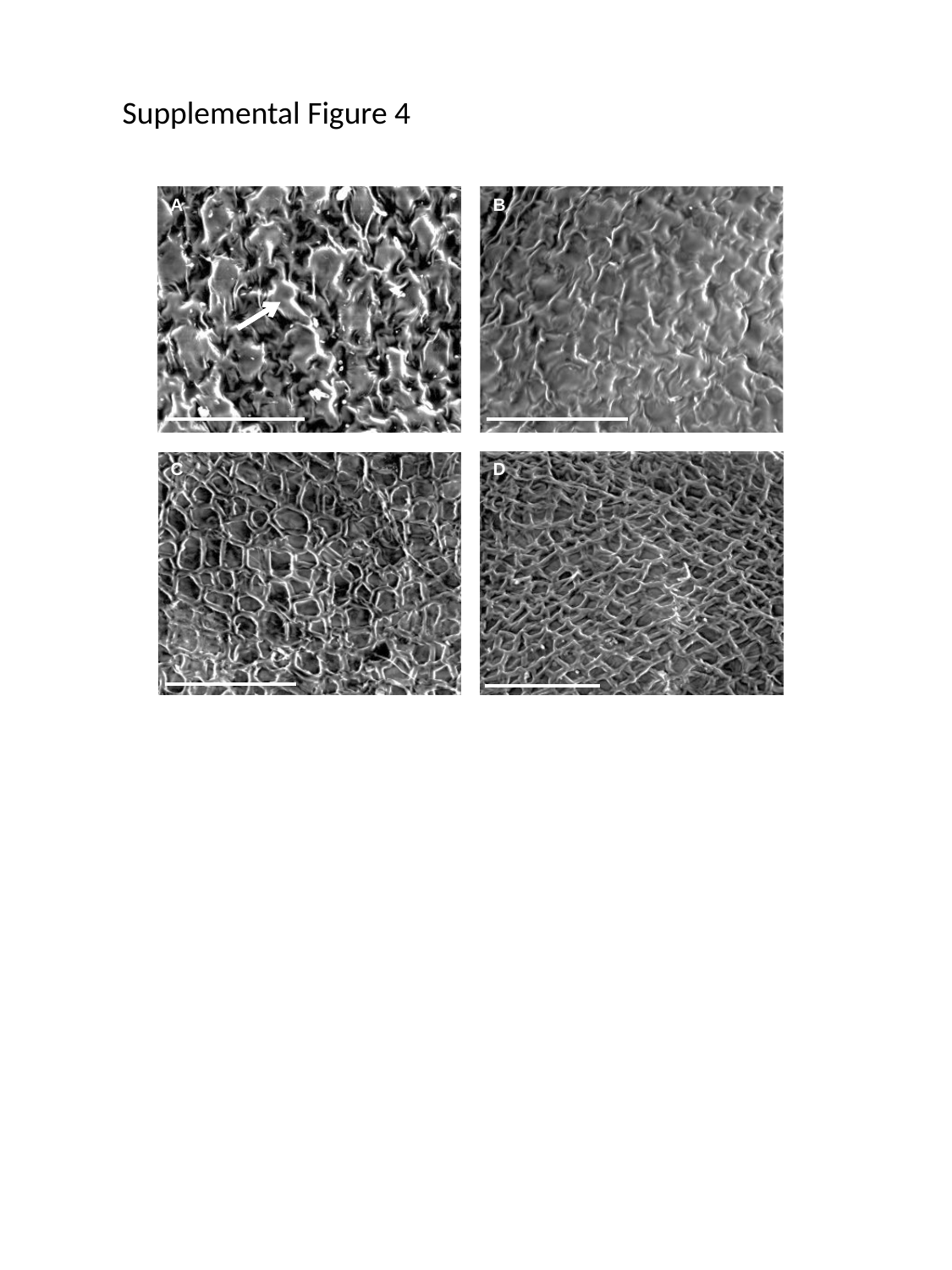

Supplemental Figure 4
A
B
D
C

Supplement: Additional file 5: Figure S4 — SEM pictures of wild type and Aattg1 mutant seeds. Scanning Electron Micrographs of the surface of A. alpina seeds. A, B) wild type Paj and pep1-1 mutant, respectively. Note, that the surface is irregularly but smooth and that the columella is seen as small domes. C, D) Aattg1-1 mutant induced in the wild type Paj background and the Aattg1-2 mutant induced in the pep1-1 background. Only the rim of the epidermal cells is left. Columellas are absent. Scale bar: 100 μm. [file 1471-2229-14-16-S5.pptx]
